# Young Women’s Ratings of Three Placebo Multipurpose Prevention Technologies for HIV and Pregnancy Prevention in a Randomized, Cross-Over Study in Kenya and South Africa

**DOI:** 10.1007/s10461-018-2078-5

**Published:** 2018-03-20

**Authors:** Alexandra M. Minnis, Sarah T. Roberts, Kawango Agot, Rachel Weinrib, Khatija Ahmed, Kgahlisho Manenzhe, Fredrick Owino, Ariane van der Straten, the TRIO Study Team

**Affiliations:** 1 Women’s Global Health Imperative, RTI International, 351 California Street, Suite 500, San Francisco, CA 94104 USA; 2 School of Public Health, University of California, Berkeley, USA; 3 Impact Research and Development Organization, Kisumu, Kenya; 4 Setshaba Research Centre, Soshanguve, South Africa; 5 Department of Medicine, Center for AIDS Prevention Studies, University of California, San Francisco, USA

**Keywords:** Multi-purpose prevention technologies, Use ratings, Acceptability, HIV prevention, End-user research, Contraception

## Abstract

End-user input is critical to inform development of multipurpose prevention technology (MPT) products that prevent HIV and pregnancy. The TRIO Study, conducted in Kenya and South Africa, enrolled 277 HIV-negative women aged 18–30 in a randomized cross-over study to use each placebo MPT (daily oral tablets, monthly injections, and monthly vaginal ring) for one month. At the end of each month, participants rated how much they liked using the product on a 5-point Likert scale (5 = liked very much). We compared mean ratings using paired t-tests and examined sociodemographic-, attribute-, and behavior-related characteristics associated with ratings using multivariable linear regression and data from in-depth interviews. After use, mean ratings were significantly higher for injections [4.3 (SD = 1.0)] compared with tablets [3.0 (SD = 1.3)] and rings [3.3 (SD = 1.4)] (p < 0.001); mean ratings for rings were significantly higher than for tablets (p = 0.013). Mean ratings of a hypothetical active MPT increased for all products after the one-month period of use, with the greatest increase for rings, the least familiar product. In multivariable analysis, acceptability of key product attributes (e.g., product look) were associated with a significant increase of ≥ 1 point in the mean rating across all three products (p ≤ 0.001). Perceived ability to use the product without partner knowledge was associated with a higher mean rating for rings (b = 0.50; p = 0.006). The acceptability of product attributes contributed significantly to the rating of all products, highlighting the value of choice in pregnancy and HIV prevention to accommodate diverse users.

## Introduction

Preventing HIV and unintended pregnancy are health priorities for women globally. In sub-Saharan Africa, nearly one in four married or in-union women are estimated to have an unmet need for contraception, with use of modern contraceptives varying across geographic settings and population groups [1]. Even in areas where contraceptive uptake is higher, method discontinuation contributes substantively to unintended births [2]. Multiple factors contribute to this unmet need, including partner influence, barriers to access, experience or fear of side effects, and inconvenience [3]. In many sub-Saharan African countries with persistent unmet need for contraceptives, women are also at high risk of HIV infection [4]. Multipurpose prevention technologies (MPTs) are an innovative class of products that deliver varied combinations of HIV prevention, other sexually transmitted infection (STI) prevention, and contraception. MPTs that combine HIV prevention and contraceptives in one delivery form offer a strategy to ease user and health systems burden as well as address key barriers to adoption of either biomedical prevention method alone [5, 6]. MPTs may increase adherence by simplifying use and capitalizing on the opportunity to integrate HIV prevention with a less stigmatized indication, pregnancy prevention.

Although MPTs already exist in the form of male and female condoms, acceptability of these products is low due to both product attributes and contextual factors. Men and women report that condoms interfere with sexual pleasure, and their use is associated with mistrust and infidelity [7–9]. New MPT products that build on successful contraceptive technologies and biomedical HIV prevention products, approved or in development, could fill a gap in the available method mix.

Placing end-users at the center of product development is now recognized as critical to the design of products that ultimately will meet the needs of target users [10–13]. End-user input is essential to informing the development of successful MPT products, including combining pre-exposure prophylaxis (PrEP) and contraception, to maximize uptake and use and ultimately, a product’s public health impact on the HIV epidemic [14]. Low adherence evidenced in recent clinical trials of HIV prevention products, related to end-user challenges and product acceptability issues, emphasizes the importance of integrating end-user perspectives early in the product development process [15–17]. Research on MPTs still in development is critical to ensure alignment between delivery forms being developed, product attributes preferred by the end-users, and, ultimately, messaging to facilitate product adoption. A variety of delivery forms (e.g., reversible; systemic) ultimately will be needed to meet the demands of different MPT market segments [10].

We conducted the TRIO Study to examine acceptability and preferences among three placebo MPT forms for prevention of HIV and unintended pregnancy among young women in Kenya and South Africa. The overall goal of this research was to obtain information that would inform decisions regarding further development of MPTs to optimize the likelihood of end-user adoption. This analysis assesses ratings of product use experiences for three delivery forms currently available for either HIV or pregnancy prevention that are candidates for an MPT product: oral tablets, injections, and vaginal rings. In addition, we used quantitative and qualitative data to examine the acceptability of product attributes and factors associated with product ratings.

## Methods

### Study Design

The TRIO Study was a randomized, mixed-methods cross-over study with two stages: a three-month cross-over period in which young women used each product for one month, followed by a two-month period in which women could choose which product to use.

The goal of TRIO was to investigate acceptability and preferences among the delivery forms; therefore, all three MPTs were placebos without any active pharmaceutical agent so we could uncouple the attributes of each delivery form from potential side effects or effectiveness. The delivery forms and use protocols are described in detail elsewhere [18]. Briefly, the MPT tablets were represented by placebo TDF/FTC with a daily dosing requirement (provided by Gilead Sciences); the MPT injections were represented by two 2 mL saline injections, one in each gluteal muscle, given at the same time once per month; and the MPT vaginal ring was represented by the silicone elastomer placebo vaginal ring developed by the International Partnership for Microbicides, which was to be used in the vagina continuously for one month.

### Participant Eligibility and Recruitment

The study was conducted at two sites: Impact Research and Development Organization (IRDO) in Kisumu, Kenya and Setshaba Research Centre (SRC) in Soshanguve, South Africa. We selected two well-established research sites where a potential MPT product would be of interest to young women—where HIV rates are high and use of modern contraceptive methods is at least moderate. To inform future MPT product development, we aimed to examine the research questions in two diverse geographic areas within sub-Saharan Africa to evaluate the consistency of findings. All study procedures received ethics and regulatory approvals prior to implementation. Participants were recruited from communities in the geographic areas served by the research sites using community mobilization and sensitization meetings, street recruitment from shopping areas, and (in South Africa only) outreach at family planning clinics and voluntary HIV counseling and testing centers [18]. Enrollment took place between December 2015 and June 2016, with follow-up visits completed in December 2016. Women were eligible for the study if they were aged 18–30 years, sexually active, non-pregnant, HIV-negative, and had never participated in HIV-prevention product trials or demonstration studies.

### Procedures

At enrollment, participants were randomized in equal numbers to one of six product-use sequences representing all possible orders of the three products [18]. Randomization was stratified by site and included blocks of size six to distribute evenly the six product sequences. Study sites received sealed, numbered randomization envelopes that were assigned in sequential order to each participant.

At enrollment, participants completed an interviewer-administered sociodemographic and product acceptability questionnaire after being introduced briefly to the products by viewing an animated educational video. At the end of the visit, they were given their first product (based on randomization sequence) with instructions for use. The site pharmacist dispensed tablets directly to participants and observed the first dose. Injections and rings were dispensed by the site pharmacist to the clinicians who then provided them to participants. Injections were administered by study clinicians. The vaginal ring was inserted by the participant at the research sites with guided instruction by study clinicians.

Each month during the cross-over period, participants completed a follow-up study visit that included an acceptability and product use questionnaire that asked about the product they had been given at their previous study visit. At follow-up visits one and two, they were given the next product in their randomization sequence. At the end of stage one (follow-up visit 3), participants were asked to choose which product they would like to use for the next two months, with an option to switch after one month. In addition, at this visit, a random sub-sample of 46 participants (23 per site with approximately equal distribution across the product sequences) completed an in-depth interview that explored product use experiences for each of the three products.

### Measures

#### Outcome

The primary outcome for this analysis was the participant’s rating of how much she liked the product after use during the previous month. The 5-point Likert scale (5 = high) corresponded to the following labeled options: dislike very much, dislike, neither like nor dislike, like, and like very much. For this analysis, the response scale was analyzed as a continuous variable. To assess changes in opinions before and after use, we compared responses to a question asked at baseline and monthly follow-up visits that assessed how much the participant would like using a hypothetical active version of each product for both pregnancy and HIV prevention, rated on the same 5-point Likert scale.

#### Predictors

We evaluated three categories of factors as potential predictors of product ratings: sociodemographic characteristics, sexual behavior, and the acceptability of product attributes. Sociodemographic characteristics included age, marital status, parity, household composition, contraceptive history and several socioeconomic indicators (employment, educational attainment and food insecurity). Sexual behaviors focused on recent behaviors in the past month (during the period of product use) and included number of partners and frequency of sexual intercourse. Product attributes assessed in common across all three products included: product look, ease of use, interference with normal activities, and discreteness. In addition, we evaluated product-specific attributes that included factors such as tablet color, dosing regimens, and ring size and thickness. Participants were asked to classify most product attributes as very unacceptable, unacceptable, acceptable, or very acceptable, and responses were collapsed into binary variables for analysis (unacceptable or acceptable). For the discreteness attribute, they were asked whether they believed the product could be used without their partner knowing and without their friends or family knowing. See Tables 2, 3 and 4 for specific measures assessed for each product.

### Analysis

Our analysis focused on the 3-month crossover period that comprised the first stage of the study. Data from each visit were analyzed according to which product the participant had used over the past month. We compared ratings of actual use between products using paired t-tests. We also examined ratings for hypothetical active versions of each product at baseline and after one month of use and assessed whether this rating changed between these two time points using a one-sample *t* test.

To estimate the association of each predictor with the rating outcome, we used linear regression models with robust standard errors. Each product (tablet, injection, and ring) was modeled separately. We examined each of the sociodemographic, sexual behavior, and attribute-related characteristics separately in bivariate models and in models adjusting for age, study site, and randomization sequence as potential confounders. We report results from the adjusted models only as there were no meaningful differences between the unadjusted and adjusted estimates.

For analysis of attribute-related characteristics associated with product ratings, we considered whether ratings were clustered (e.g., did women rate all attributes as acceptable or unacceptable). To evaluate this, we calculated pairwise correlations among attributes and the total number rated as acceptable. Most product attribute ratings were not collinear or highly clustered. Only two sets of attributes had correlations > 0.5: the acceptability of the number of injections received at a given time and of having had 2 injections in a month (rho = 0.53) and the acceptability of how the ring felt during sex to the participant and to her partner (rho = 0.79).

We coded in-depth interview transcripts using a codebook informed by our team’s past work in similar studies [19] and a conceptual model of HIV prevention product acceptability [20]. A team of three analysts coded all transcripts using Dedoose, a web-based qualitative analysis software. Inter-rater reliability scores were 0.82 (pooled Cohen’s kappa). To elucidate quantitative findings in this paper, we reviewed code reports pertaining to product attributes that aligned with the core quantitative measures assessed in common across the three products: product look, ease of use, interference with normal activities, and discreteness. We drew excerpts from interviews with participants who found the respective feature of a given product to be “acceptable” based on the quantitative data. In addition, to explore changes in the ratings of a hypothetical active version of each product, we examined code reports that addressed changes in acceptability and preferences after product use.

## Results

### Study Population Characteristics

The study enrolled 277 participants; our analytical sample consists of the 258 participants (93.1%) who completed at least one follow-up visit during the cross-over period: 255 participants reported on tablet use and 254 reported on injection and ring use. As presented in Table 1, two-thirds (67.1%) of participants were aged 18–24 years; few (25.6%) were married; most (79.5%) had been pregnant previously; and half (51.9%) had completed secondary school. Socioeconomic indicators varied between sites, with South African women reporting higher educational attainment and lower levels of marriage and food insecurity than Kenyan participants. Past use of modern contraceptives was similar across sites, though a higher proportion of women in South Africa than Kenya had used contraceptive injections (81.5% vs. 59.4%, p < 0.001) and a higher proportion of women in Kenya had used contraceptive implants or IUDs (47.7% vs. 30.0%, p = 0.004).

**Table 1:** Baseline participant characteristics, TRIO Study, 2015–2016

N^a^	Total	South Africa	Kenya
	n (%)	n (%)	n (%)
	258 (100.00)	130 (100.00)	128 (100.00)
Age			
18–24	173 (67.1)	87 (66.9)	86 (67.2)
25–30	85 (33.0)	43 (33.1)	42 (32.8)
Married or cohabiting	66 (25.6)	5 (3.9)	61 (47.7)
Pregnancy history (≥ 1)	205 (79.5)	102 (78.5)	103 (80.5)
Completed secondary school	134 (51.9)	82 (63.1)	52 (40.6)
Earns an income	82 (31.9)	18 (13.9)	64 (50.4)
Lives with			
Parents or grandparents	132 (52.6)	103 (79.2)	29 (22.7)
Husband/boyfriend	70 (27.9)	10 (7.7)	60 (46.9)
Other	49 (19.5)	17 (13.1)	39 (30.5)
Food insecurity, past 4 weeks			
Never	118 (45.7)	81 (62.3)	37 (28.9)
Rarely or sometimes	101 (39.2)	32 (24.6)	69 (53.9)
Often	39 (15.1)	17 (13.1)	22 (17.2)
Has privacy in the home	213 (82.9)	119 (92.3)	94 (73.4)
# sex partners, past 30 days			
None	12 (4.7)	2 (1.5)	10 (7.8)
One	216 (83.7)	118 (90.8)	98 (76.6)
More than one	30 (11.6)	10 (7.7)	20 (15.6)
Ever used injectables	182 (70.5)	106 (81.5)	76 (59.4)
Ever used oral contraceptives	67 (26.0)	31 (23.9)	36 (28.1)
Ever used implant or IUD	100 (38.8)	39 (30.0)	61 (47.7)
Any vaginal insertion, past 3 months^b^	170 (65.9)	102 (78.5)	68 (53.1)

^a^Includes all women with at least 1 product rating during follow-up

^b^Includes use of any of the following inside the vagina for menstrual control: tissue, toilet paper, cotton wool, cloth, tampon, water with or without soap; or any of the following inside the vagina when not on menses: water with or without soap, fingers, paper, cloth, cotton wool, or materials to dry or tighten the vagina

### Product Ratings After One Month of Use

Product ratings after one month of use were highest for injections (mean 4.26; 95% CI 4.14, 4.38), followed by rings (mean 3.28; 95% CI 3.11, 3.45) and then tablets (mean 2.96; 95% CI 2.81, 3.12). The mean rating for injections was significantly higher than those for rings and tablets (p < 0.001); the mean rating for rings was also significantly higher than that for tablets (p = 0.015).

### Ratings of a Hypothetical Active Product Before and After One Month of Use

**Fig. 1:**
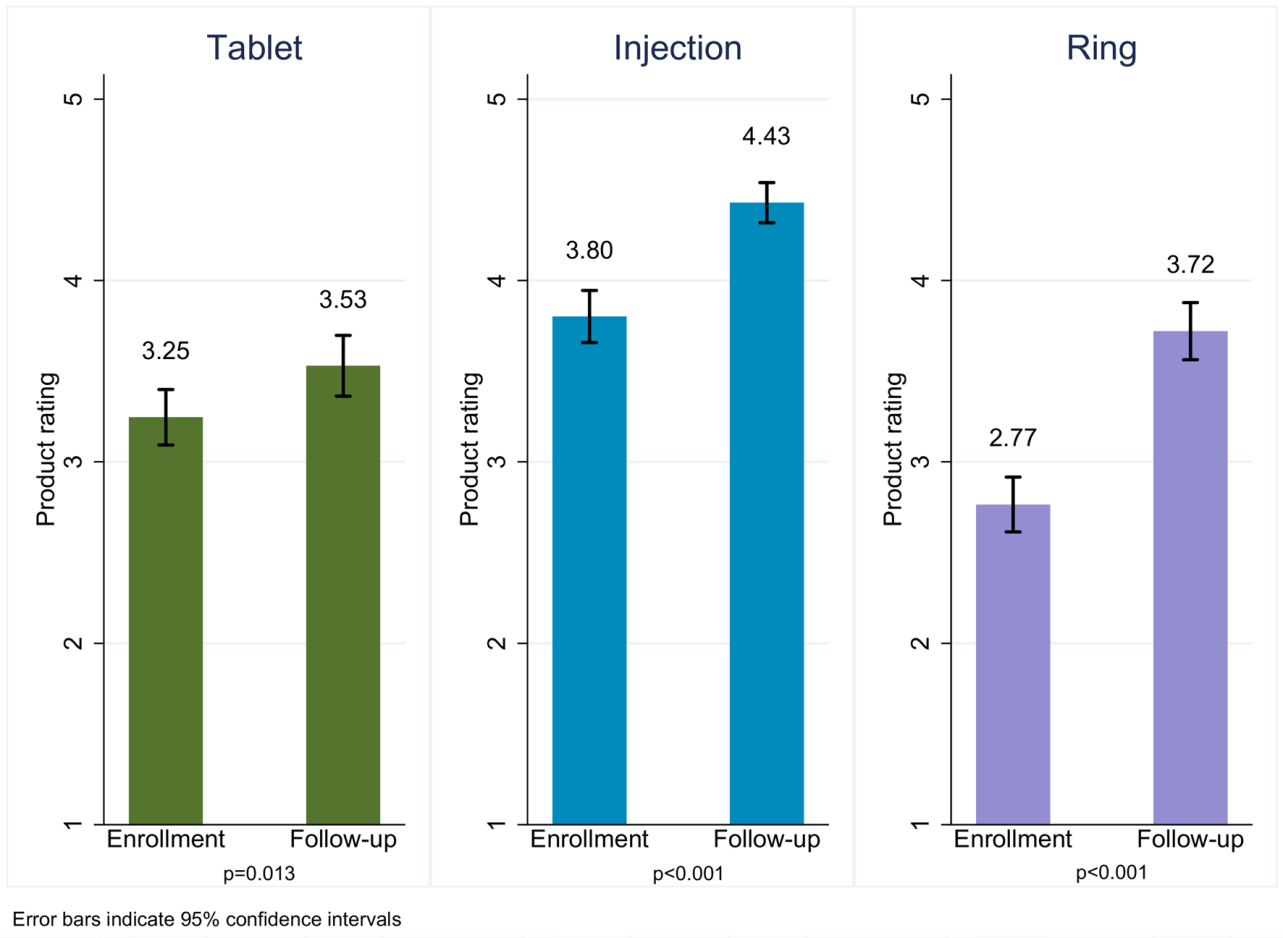
Ratings of a hypothetical active MPT product at enrollment and during cross-over period

Mean ratings, at enrollment, of a hypothetical active version of each product were highest for injections (mean 3.80; 95% CI 3.66, 3.95), followed by tablets (mean 3.25; 95% CI 3.09, 3.40), and then ring (mean 2.77; 95% CI 2.61, 2.92) (Fig. 1). For all three products, the mean product ratings increased significantly from enrollment after one month of use. The increase was greatest for rings (mean of 0.97 points; 95% CI 0.75, 1.16; p < 0.001), followed by injections (mean of 0.62 points; 95% CI 0.45, 0.79; p < 0.001) and then tablets (mean of 0.24 points (95% CI 0.05, 0.42; p = 0.013) (Fig. 1). After 1 month of use, injections remained the most highly rated, but rings shifted from an average rating of below neutral (value of 3.0) to a mean of 3.72. During follow-up, mean ratings were higher for the hypothetical active product than for product used for the past month, reflecting the difference between active and placebo product.

In-depth interview data illustrate user experiences that underlie the increases in ratings found for each of the three products. Changes in tablet acceptability were frequently described as occurring because participants became accustomed to physical attributes (i.e., size, color) that, at first, they disliked or even found “shocking.” One woman’s comments about the tablet echoed remarks made by others, “I was scared the first time I saw the tablets. I was just—wow, this tablet is big, but the swallowing was not a problem.” (South Africa, age 21) In discussing injections, past experience with injectable contraceptives typically allayed negative opinions at the outset. For those women who did express negative reactions to the injection, they discussed being afraid of pain with the injections when they first saw the needle; however, these feelings subsided after receiving them, with comments from multiple participants that this product was “a once-off thing” and “nothing to worry about.” Many women described the opportunity to try the ring and gain familiarity with it as critical to improved acceptability. This participant illustrates this shift when describing how she felt the first time saw the ring and how swiftly her opinion changed after trying the product: “I was like wow!! This aah circle thing, ring vaginal is going to enter my vagina?! I was like argh!! Like I was thinking about pains and what…. I thought I won’t be comfortable but I was comfortable you know. After one day, aah!! I was like wow, I don’t feel nothing. It’s like normal.” (South Africa, age 26).

### Predictors of Product Rating After Use

Few sociodemographic or behavioral characteristics were significantly associated with product ratings. Women in Kenya rated tablets higher and rings lower than women in South Africa (tablets: adjusted β 0.45; 95% CI 0.13, 0.76; p = 0.006; rings: adjusted β − 0.35; 95% CI − 0.66, 0.01, p = 0.058), while site was not associated with injection ratings (Tables 2, 3, 4). Participants who had completed secondary school reported a lower mean rating for tablets compared with those who had not completed secondary school (adjusted β − 0.38; 95% CI − 0.71, − 0.06; p = 0.021), and a higher mean rating for injections (adjusted β 0.24, 95% CI 0.00, 0.49, p = 0.054). Ever-use of injectable contraceptives was associated with slightly lower injection ratings (adjusted β − 0.24, 95% CI − 0.48, 0.00, p = 0.054). Though prior use of injectable contraceptives and implants varied between Kenya and South Africa, the associations between contraceptive method use and product ratings did not vary by site. There was a small but statistically significant association between the level of sexual activity and ring ratings: for each additional sex act reported in the past month, the ring was rated 0.04 points higher (95% CI 0.00, 0.07; p = 0.025). Having privacy in the home and frequent food insecurity appeared to be influential to ring rating, but did not reach statistical significance at p < 0.05.

**Table 2:** Predictors of tablet ratings after one-month of use (N = 255 visits)

	N (%)	β^a^	95% CI	p value
Baseline participant characteristics				
Age				
18–24	173 (67.8)	Ref		
25–30	82 (32.2)	0.02	− 0.32, 0.35	0.928
Site				
South Africa	128 (50.2)	Ref		
Kenya	127 (49.8)	0.45	0.13, 0.76	0.006
Married or cohabiting	66 (25.9)	− 0.13	− 0.54, 0.28	0.537
Ever pregnant	202 (79.2)	0.00	− 0.42, 0.43	0.996
Completed secondary school	134 (52.6)	− 0.38	− 0.71, − 0.06	0.021
Earns an income	82 (32.3)	− 0.11	− 0.48, 0.27	0.578
Lives with				
Parents or grandparents	131 (51.4)	Ref		
Husband/boyfriend	69 (27.1)	− 0.28	− 0.73, 0.16	0.213
Other	55 (21.6)	− 0.23	− 0.66, 0.21	0.310
Food insecurity				
Never	116 (45.5)	Ref		
Rarely or sometimes	100 (39.2)	− 0.12	− 0.49, 0.25	0.520
Often	39 (15.3)	0.03	− 0.45, 0.52	0.887
Has privacy in the home	210 (82.7)	0.24	− 0.19, 0.68	0.276
Ever used oral contraceptives	67 (26.3)	0.12	− 0.22, 0.46	0.473
Sexual behavior				
# sex acts, past month (mean, SD)	5.02 (4.6)	0.01	− 0.03, 0.04	0.600
# sex partners, past month				
0 or 1	227 (89.0)	Ref		
> 1	28 (11.0)	0.29	− 0.21, 0.79	0.254
Product attributes				
Reports the following attribute as acceptable (vs. unacceptable)				
Product look	157 (61.6)	1.06	0.72, 1.40	< 0.001
Ease of use	149 (58.4)	1.15	0.85, 1.45	< 0.001
Interference with normal activities	170 (66.7)	1.16	0.82, 1.49	< 0.001
Tablet color	190 (74.5)	0.70	0.32, 1.08	< 0.001
How it felt to swallow the tablets	128 (50.2)	0.95	0.64, 1.26	< 0.001
How stomach felt after taking tablets	186 (72.9)	0.92	0.57, 1.27	< 0.001
Taking a tablet every day	132 (51.8)	1.32	1.05, 1.6	< 0.001
How the tablet felt in hands	180 (70.6)	0.84	0.48, 1.2	< 0.001
Size of tablets	99 (38.8)	0.64	0.32, 0.95	< 0.001
Possible to use without partner knowledge	176 (69.0)	0.19	− 0.17, 0.55	0.297
Possible to use without family knowledge	167 (65.5)	0.17	− 0.19, 0.53	0.358

^a^Adjusted for age, study site (South Africa vs. Kenya), and randomization sequence

**Table 3:** Predictors of injection ratings after one-month of use (N = 254 visits)

	N (%)	β^a^	95% CI	p value
Baseline participant characteristics				
Age				
18–24	169 (66.5)	Ref		
25–30	85 (33.5)	0.08	− 0.20, 0.35	0.578
Site				
South Africa	129 (50.8)	Ref		
Kenya	125 (49.2)	− 0.04	− 0.29, 0.21	0.743
Married or cohabiting	65 (25.6)	− 0.13	− 0.42, 0.17	0.405
Ever pregnant	202 (79.5)	− 0.16	− 0.44, 0.13	0.281
Completed secondary school	133 (52.4)	0.24	0.00, 0.49	0.054
Earns an income	79 (31.2)	− 0.05	− 0.34, 0.25	0.755
Lives with				
Parents or grandparents	129 (50.8)	Ref		
Husband/boyfriend	69 (27.2)	− 0.26	− 0.62, 0.11	0.172
Other	56 (22.1)	− 0.06	− 0.41, 0.29	0.744
Food insecurity				
Never	117 (46.1)	Ref		
Rarely or sometimes	99 (39.0)	0.00	− 0.28, 0.27	0.990
Often	38 (15.0)	0.08	− 0.32, 0.48	0.688
Has privacy in the home	210 (83.0)	0.02	− 0.27, 0.32	0.866
Ever used injectable contraceptives	179 (70.5)	− 0.24	− 0.48, 0.00	0.054
Sexual behavior				
# sex acts, past month (mean, SD)	4.85 (5.4)	0.01	− 0.01, 0.03	0.314
# sex partners, past month				
0 or 1	222 (87.4)	Ref		
> 1	32 (12.6)	− 0.17	− 0.68, 0.34	0.509
Product attributes				
Reports the following attribute as acceptable (vs. unacceptable)				
Product look	224 (88.2)	1.11	0.62, 1.59	< 0.001
Ease of use	236 (92.9)	1.10	0.48, 1.73	0.001
Interference with normal activities	230 (90.6)	1.11	0.55, 1.67	< 0.001
How the needle felt	157 (61.8)	0.43	0.16, 0.70	0.002
Feeling at injection site 1 day later	217 (85.4)	0.79	0.41, 1.18	< 0.001
Number of injections at a time	178 (70.1)	0.41	0.11, 0.71	0.008
Having 2 injections per month	171 (67.3)	0.55	0.24, 0.86	0.001
Possible to use without partner knowledge	198 (78.0)	0.07	− 0.28, 0.41	0.702
Possible to use without family knowledge	186 (73.2)	− 0.11	− 0.42, 0.19	0.460

^a^Adjusted for age, study site (South Africa vs. Kenya), and randomization sequence

**Table 4:** Predictors of ring ratings after one-month of use (N = 254 visits)

	N (%)	β^a^	95% CI	p value
Baseline participant characteristics				
Age				
18–24	171 (67.3)	Ref		
25–30	83 (32.7)	0.25	− 0.10, 0.61	0.165
Site				
South Africa	127 (50.0)	Ref		
Kenya	127 (50.0)	− 0.32	− 0.66, 0.01	0.058
Married or cohabiting	66 (26.0)	− 0.32	− 0.81, 0.17	0.204
Ever pregnant	201 (79.1)	− 0.02	− 0.45, 0.41	0.204
Completed secondary school	133 (52.4)	− 0.30	− 0.66, 0.06	0.099
Earns an income	80 (31.6)	0.15	− 0.26, 0.56	0.477
Who she lives with				
Parents or grandparents	129 (50.8)	Ref		
Husband/boyfriend	69 (27.2)	− 0.40	− 0.94, 0.15	0.151
Other	56 (22.1)	− 0.41	− 0.87, 0.06	0.085
Food insecurity				
Never	116 (45.7)	Ref		
Rarely or sometimes	100 (39.4)	0.19	− 0.21, 0.58	0.351
Often	38 (15.0)	0.47	− 0.03, 0.97	0.066
Has privacy in the home	209 (82.6)	0.39	− 0.05, 0.84	0.085
Ever used IUD or implant	98 (38.6)	0.25	− 0.1, 0.59	0.157
Any vaginal insertion, past 3 months	166 (65.4)	− 0.20	− 0.56, 0.16	0.279
Sexual behavior				
# sex acts, past month (mean, SD)	4.39 (4.8)	0.04	0.00, 0.07	0.025
# sex partners, past month				
0 or 1	232 (91.3)	Ref		
> 1	22 (8.7)	0.26	− 0.35, 0.87	0.403
Product attributes				
Reports the following attribute as acceptable (vs. unacceptable)				
Product look	169 (66.5)	1.36	1.02, 1.70	< 0.001
Ease of use	189 (74.4)	1.81	1.47, 2.14	< 0.001
Interference with normal activities	186 (73.2)	1.45	1.11, 1.79	< 0.001
Ring size	156 (61.4)	0.80	0.46, 1.14	< 0.001
Inserting the ring	162 (63.8)	0.78	0.41, 1.14	< 0.001
Removing the ring	179 (72.5)	0.53	0.12, 0.94	0.011
How the ring felt during sex	142 (55.9)	1.36	1.01, 1.7	< 0.001
How the ring felt to partner during sex	151 (59.7)	1.12	0.74, 1.49	< 0.001
How the ring felt during menses	137 (53.9)	0.93	0.52, 1.34	< 0.001
Leaving in ring for entire month	180 (71.7)	1.68	1.35, 2.00	< 0.001
How the ring felt in hands	156 (61.7)	0.58	0.24, 0.93	0.001
Possible to use without partner knowledge	159 (62.6)	0.49	0.14, 0.84	0.006
Possible to use without family knowledge	204 (80.3)	− 0.01	− 0.45, 0.44	0.981

^a^Adjusted for age, study site (South Africa vs. Kenya), and randomization sequence

Product attributes were more strongly associated with product ratings than sociodemographic or behavioral factors. The overall acceptability of the attributes assessed in common varied across the three products, with, for example, 61.8% of women finding the “look” of the tablet acceptable, compared with 88.2% for the injection and 66.5% for the ring (Tables 2, 3, 4). Likewise, perceived interference with normal activities varied, with 66.7% reporting the level of interference associated with tablet use as acceptable, compared with 90.6% for injections and 73.2% for the ring. Across all three products, reporting the product look, ease of use and interference with normal activities as acceptable were each associated with higher mean ratings (p ≤ 0.001). The magnitude of the increases in ratings associated with each attribute was highest for rings. For instance, ring rating was 1.81 points higher (95% CI 1.47, 2.14) among women who found the product’s “ease of use” acceptable, compared with those women who found it not acceptable. In comparison, this increase was 1.10 points (95% CI 0.48, 1.73) for injections and 1.15 points for tablets (95% CI 0.85, 1.45). Unlike for tablets and injections, the ability to use the product covertly appeared influential only to ring rating (Table 4). Women who reported it was possible for them to use the ring without their partner knowing (62.6% of respondents) reported a higher mean rating of the ring (adjusted β 0.49; 95% CI 0.14, 0.84; p = 0.006) than those who reported it was not possible.

For all three products, multiple product-specific attributes were associated with product rating after use. Of those assessed for tablets, the least acceptable were how it felt to swallow tablets (50.2%), daily tablet taking (51.8%) and tablet size (38.8%). The magnitude of the estimated increase in tablet rating for each tablet-specific attribute ranged from 0.64 to 1.32 points (all p < 0.001). For injections, in general, the level of acceptability of product-specific attributes was higher than for the other products (Table 3). However, the effects of most product-specific attributes (e.g., how the needle felt, the feeling at the injection site one day later) on injection rating were more modest (increases in rating ranged from 0.43 to 0.79 points). The acceptability of numerous ring-specific attributes (e.g., ring size, insertion and removal, how the ring felt during sex) were significantly associated with increases in the rating of the ring. The most influential ring-specific attribute to rating was the acceptability of leaving the ring inserted for the entire month (adjusted β 1.68; 95% CI 1.35, 2.00; p < 0.001) while the least influential was the acceptability of removing the ring (adjusted β 0.53, 95% CI 0.12, 0.94; p < 0.001). Reporting as acceptable how the ring felt during sex, both for the participant and for her partner, was associated with a greater than one-point increase in mean rating of the ring.

### Qualitative Findings Pertaining to Attributes and Preferences

In reflecting on product attributes that affected overall use experiences, participants discussed physical features of the products that were central to acceptability and ease of use. Among participants who found that general physical attributes of the tablet were acceptable overall, tablet size was nonetheless raised frequently as a persistent concern. Tablets were regarded by many women as large and intimidating to swallow and, while some indicated they became accustomed to swallowing them, others said the size remained a barrier to use. For those TRIO participants who found the physical attributes of the injection to be acceptable, there was far less discussion of these aspects in comparison to the other two products. These participants primarily described that the size of the needle scared them, as articulated by this South African participant, “I must admit the injection, oh no! The needle! It’s a bit huge” (South Africa, age 21). However, this aversion was primarily described as a visual reaction rather related to the experience of receiving the injection itself. Among women who found that physical attributes of the ring were acceptable, the most commonly discussed physical attribute was its size. In general, these participants seemed to react to the diameter more than the thickness of the ring—for those who thought the ring was too large, the size seemed to be primarily a visual or psychological barrier rather than an experiential one. Even though a number of these participants indicated that once they inserted the ring they did not feel it, they nonetheless suggested that it be made smaller because the “idea” of keeping such a large thing inside their bodies was disconcerting. As one participant described, “I think that if some people see the ring then they see the size and say that this thing is so big that I cannot use. So what I would want to tell them [product developers] is that they reduce the size” (Kenya, age 21).

Participants who found products acceptable based on several dimensions of use attributes—ease of use, interference with normal activities and discreetness—offered reflections on product characteristics that affected use experiences and perceived burden. TRIO participants who reported the tablets’ ease of use as acceptable, for example, still maintained reservations about the tablets when discussing them during in-depth interviews. They described persistent barriers to easy use of the tablets, including negative sensations when swallowing tablets, challenges with discreetness and difficulty with adhering to a daily dosing regimen. As one woman who resided in a one-room house noted, “It is difficult to take the drug in front of the visitor because they might go saying things that you don’t know” (Kenya, age 24). Women who indicated the injection’s attributes tied to use were acceptable very consistently expressed positive views of the product, valuing the fact that it “saved time” and offered discreetness. One participant described, “after being injected, you are done and [do] not keep using it every day. You just leave because it’s not like other drugs that you have to go home with. It’s something that you get injected and it’s over. You just leave the clinic the way you came.” (Kenya, age 18). Participants who indicated that ease of use of the ring was “acceptable” defined this in several ways, including whether they could detect it once inserted; whether it caused physical discomfort, particularly during insertion or removal; and whether they experienced difficulty with ring insertion. One South African woman described that she liked that “when [the ring is] already inside you, it stays leveled. It doesn’t move, it doesn’t do anything. It just stays where you put it” (South Africa, age 21). Others, as reflected in this comment by a Kenyan woman, conceptualized ease of use as discreetness, “I liked it because once you have inserted it, you are just okay. No one knows that you have inserted something in you…no one knows, it is private” (Kenya, age 25). Though many women expressed that they had initial concerns that their partners might feel the ring during sex, few actually noted that their partner did, in fact, detect the ring.

## Discussion

We found that when given the opportunity to try three placebo MPT products, after using the products for one month, women in the TRIO study rated injections most highly, followed by rings, and then tablets. The mean rating for injections fell between “like” and “like very much” on the 5-point Likert scale, while the ratings for rings and tablets were slightly above and below the “neither like nor dislike” category, respectively. With an opportunity to use the products for one month each, for all products, the rating of a hypothetical active MPT product increased from enrollment. This was particularly the case for the ring, a product with which women had no familiarity.

These findings highlight the varying experiences young women had with the three delivery forms examined in the TRIO Study, and point to an overall desire for longer-acting products, with injections emerging as a clear favorite at both time points. Product rating findings align with the preferences indicated through product choice by participants for use during stage two of the study, though, despite the ring being rated higher than tablets in this analysis, product choice for use during stage two of the study did not differ significantly between the two [21]. The increase in ratings of a hypothetical active MPT ring after use suggests that, for some women, initial perceptions were influenced by a lack of familiarity with this dosage form, and that end-user preference for this product may increase with increased opportunity to try it and gain experience with some of the physical attributes that, at first, appeared as barriers to use. As MPT intravaginal rings move forward in development, consideration of strategies to increase familiarity with rings, including through ongoing HIV PrEP demonstration projects will be critical to wider acceptance and ultimately adoption of an effective product. The product that is currently available for HIV prevention—tablets—was the lowest rated, highlighting the ongoing need for additional methods for prevention. Offering diverse choices to women, supported by education and engagement of communities and health providers, is likely critical to optimizing the adoption of MPT products. Indeed, contraceptive research underscores that uptake and use is higher with an expanded method mix to meet different user needs and to offer prevention products that are well-matched with distinct points in the reproductive life-course [22]. Furthermore, a range of prevention choices will support individuals in using products that align with periods of risk over time [23].

Product attributes were most influential on product ratings. Attributes related to user burden, such as ease of use and dosing regimen, had the greatest effects on mean ratings. Several social factors we hypothesized to be important, such as partner characteristics and household structure, were not associated with product rating. The ability to use the ring without a partner’s knowledge was an important predictor of ring rating, echoing findings from other vaginal ring studies in sub-Saharan African populations [19]. Ring acceptability was also influenced by the frequency of sexual activity participants reported. In the ASPIRE vaginal ring study, women reported that they were initially worried that the ring may cause discomfort during sex or that it may be discovered by male partners, but that these worries abated over time, and some women attributed increased sexual pleasure to the ring [19, 24]. In TRIO, more experience with sex during the one month of ring use, alongside finding that it was possible to use the ring discreetly without partner knowledge, may have helped reduce concerns and increase comfort, thereby increasing overall acceptability. Indeed, reporting as acceptable how the ring felt during sex (both to the participant and her partner) was associated with an increase in mean rating for the ring.

Several factors related to study design should be considered when interpreting the findings. TRIO was designed with placebo products to permit examination of preferences among delivery forms, first and foremost, without consideration of side effects and adverse events that might result from use of active products. Side effects do constitute an important contributor to contraceptive and PrEP discontinuation [25, 26] and would likely contribute to uptake and adherence of a future MPT. Discussions of product dosing and drug exposure were infrequently mentioned by participants, likely owing to our use of placebo products. The one-month period of use during the cross-over phase provided an opportunity for participants to try each product but does not mimic sustained use; product satisfaction ratings and preferences could shift with extended use. Finally, the monthly dosing period may not reflect the eventual active product regimen, with longer-lasting injections and rings currently under development. Nonetheless, preferred dosing frequency for long-acting products is a question that warrants further examination. However, this study design afforded an opportunity for participants to try using each product during stage one of the study and then select one to use for a more extended time period during stage two.

## Conclusions

The TRIO Study offers a unique examination of potential MPTs for HIV and pregnancy prevention based on young women’s actual use of three placebo products. The study engaged women as “co-designers” who reported on product use and acceptability, informed by having tried each product. Information about modifiable product attributes that are most influential to acceptability is valuable during product development to maximize the chance that a new product ultimately will be liked and adopted by women. The importance of product attributes to product ratings and acceptability, more than sociodemographic and contextual factors, suggests that physical features of the products themselves as well as low burden assume key roles in shaping acceptability [24]. As evidenced through multiple clinical trials of oral pre-exposure prophylaxis for HIV prevention, variations in efficacy are shaped largely by adherence [27]; adherence is often lowest among young women [28]. The findings from this study emphasize the urgency of developing HIV and pregnancy prevention products that minimize user burden. Combining both pregnancy and HIV prevention indications in an MPT would empower women with a tool to better protect their health and control their fertility.
